# Limited Predictive Value of NLR, PLR and SII in Thrombosed Hemorrhoids: A Real-World Analysis

**DOI:** 10.3390/life16040568

**Published:** 2026-03-31

**Authors:** Liviu Vasile, Stelian-Stefaniță Mogoantă, Tiberiu-Ștefăniță Țenea-Cojan, Nicolae-Dragoș Mărgăritescu, Laurențiu Augustus Barbu

**Affiliations:** 1 Department of Surgery, Emergency County Hospital, University of Medicine and Pharmacy of Craiova, 2 Petru Rares Street, 200349 Craiova, Romania; vliviu777@yahoo.com (L.V.); ssmogo@yahoo.com (S.-S.M.); 2 Department of Surgery, Railway Clinical Hospital Craiova, University of Medicine and Pharmacy of Craiova, 2 Petru Rares Street, 200349 Craiova, Romania; tiberiu.tenea@umfcv.ro (T.-Ș.Ț.-C.); laurentiu.barbu@umfcv.ro (L.A.B.)

**Keywords:** hemorrhoidal disease, thrombosed hemorrhoids, neutrophil-to-lymphocyte ratio, platelet-to-lymphocyte ratio, systemic immune inflammation index, inflammatory biomarkers, real-world study

## Abstract

**Background:** Systemic inflammatory indices such as the neutrophil-to-lymphocyte ratio (NLR), platelet-to-lymphocyte ratio (PLR) and systemic immune inflammation index (SII) have been increasingly investigated as inexpensive biomarkers in various inflammatory and thrombotic conditions. However, their clinical utility in thrombosed hemorrhoids remains poorly defined. This study aimed to evaluate the diagnostic and predictive performance of NLR, PLR and SII in a real-world cohort of patients with hemorrhoidal disease. **Methods:** We conducted a retrospective observational study including 120 consecutive adult patients admitted with hemorrhoidal disease between January 2020 and December 2024. Systemic inflammatory indices were calculated from admission blood tests. Comparative analyses, receiver operating characteristic (ROC) curves, tertile trend assessment, and univariate and multivariable logistic regression models were used to evaluate the association between inflammatory indices and thrombosed hemorrhoids. **Results:** Thrombosed hemorrhoids were identified in 55.8% of patients. Median values of NLR, PLR and SII did not differ significantly between thrombosed and non-thrombosed cases (all *p* > 0.05). ROC analysis demonstrated poor discriminatory performance (AUC: 0.52 for NLR, 0.55 for PLR, and 0.51 for SII). In multivariable analysis adjusted for age, sex and bleeding status, none of the inflammatory indices were independently associated with thrombosed hemorrhoids (all *p* > 0.05). No significant trends were observed across NLR tertiles. **Conclusions:** In this real-world cohort, systemic inflammatory indices showed limited diagnostic and predictive value for thrombosed hemorrhoids. These findings suggest that routine inflammatory ratios provide minimal incremental benefit beyond standard clinical assessment in this predominantly localized benign anorectal condition. Further prospective multicenter studies exploring dynamic and tissue-level biomarkers are warranted.

## 1. Introduction

Hemorrhoidal disease (HD) is one of the most common anorectal conditions worldwide, with a reported prevalence ranging between 11% and 39%, representing a substantial burden on healthcare systems and patients’ quality of life [1,2]. Despite its high frequency, the etiopathogenesis of hemorrhoidal disease remains incompletely understood and continues to be an area of active investigation [3].

From a pathophysiological perspective, hemorrhoids represent the symptomatic enlargement and distal displacement of normal anal cushions, structures that play an important role in fine continence [4]. Clinical manifestations typically include painless rectal bleeding, prolapse, discomfort and pruritus, with bleeding being among the most frequent presenting symptoms [5]. Although most cases follow a benign course, complications such as acute thrombosis may lead to significant pain and require urgent clinical assessment.

Increasing evidence suggests that hemorrhoidal disease is not solely a mechanical or vascular disorder but also involves active inflammatory processes. Recent prospective clinical data further emphasize that disease severity and local pathological changes play a dominant role in hemorrhoidal outcomes, supporting the concept of a predominantly compartmentalized pathophysiological mechanism [6]. Histopathological studies have demonstrated lymphocytic infiltration and increased production of pro-inflammatory mediators, including interleukin-6, tumor necrosis factor-α, nitric oxide and prostaglandin E2, supporting the presence of local immune activation [7,8]. Furthermore, vascular abnormalities such as venous dilatation, endothelial dysfunction, and degenerative changes in the supporting connective tissue have been described, contributing to disease progression and thrombotic complications [2,9]. More recently, genetic and epigenetic mechanisms, including microRNA-mediated regulation, have been implicated in the inflammatory and vascular remodeling pathways involved in hemorrhoidal disease [7,10,11].

In parallel, systemic inflammatory indices derived from routine blood tests—such as the neutrophil-to-lymphocyte ratio (NLR), platelet-to-lymphocyte ratio (PLR) and systemic immune inflammation index (SII)—have gained increasing attention as inexpensive and readily available biomarkers reflecting the host inflammatory status. Systemic inflammatory indices derived from routine blood tests have been widely investigated as prognostic biomarkers in colorectal and oncologic diseases [12]. These indices have shown diagnostic and prognostic value in a variety of inflammatory and neoplastic conditions, particularly in colorectal pathology [8,13,14]. However, their role in benign anorectal disorders remains less clearly defined.

Limited data suggest that elevated inflammatory markers may be associated with adverse outcomes in patients undergoing hemorrhoid surgery, but evidence regarding their ability to identify or predict thrombosed hemorrhoids is scarce and inconsistent [15,16]. Moreover, most available studies have evaluated isolated markers or highly selected populations, and real-world analyses integrating multiple inflammatory indices are lacking [17].

Therefore, the aim of the present study was to evaluate the diagnostic and predictive performance of NLR, PLR and SII in patients with thrombosed hemorrhoids in a real-world clinical cohort and to determine whether these readily available hematological indices provide meaningful additional information beyond standard clinical assessment.

## 2. Materials and Methods

### 2.1. Study Design and Population

This retrospective observational study included consecutive adult patients admitted with hemorrhoidal disease to the Departments of Surgery I, II and III of the County Clinical Emergency Hospital Craiova between January 2020 and December 2024. Eligible patients were identified through the institutional electronic medical records database. Only patients with confirmed hemorrhoidal disease and complete admission blood count data were included. Inclusion criteria were: (1) age ≥ 18 years; (2) confirmed diagnosis of hemorrhoidal disease based on clinical examination; and (3) availability of complete blood count parameters at presentation. Exclusion criteria included: (1) active systemic infection; (2) known inflammatory or autoimmune disease; (3) hematologic malignancy; (4) current immunosuppressive therapy; and (5) incomplete laboratory data. These exclusion criteria were applied to minimize the influence of systemic inflammatory conditions and treatments that could affect hematological indices. All patients included in the study were hospitalized cases, as data were derived exclusively from the surgical inpatient database.

A total of 120 patients met the eligibility criteria and were included in the final analysis.

### 2.2. Clinical Assessment

All patients underwent standard proctologic evaluation performed by experienced colorectal surgeons. Hemorrhoid phenotype (internal, external, or mixed), presence of thrombosis, and active bleeding were recorded at admission.

Thrombosed hemorrhoids were diagnosed clinically at admission by experienced surgeons during standard proctologic examination. The diagnosis was based on the presence of acute painful, tense perianal swelling with characteristic bluish discoloration on physical examination. In this study, thrombosis predominantly referred to external hemorrhoidal thrombosis, as typically encountered in acute clinical presentations. Bleeding status was determined based on patient history and direct clinical assessment at presentation. Demographic data, length of hospital stay, and relevant clinical variables were extracted from the electronic medical records.

### 2.3. Laboratory Measurements and Calculation of Inflammatory Indices

Peripheral venous blood samples were obtained at hospital presentation prior to any therapeutic intervention and analyzed using standard automated hematology analyzers in the hospital laboratory.

The following hematological parameters were recorded:

- white blood cell count
- absolute neutrophil count
- absolute lymphocyte count
- platelet count

Systemic inflammatory indices were calculated using baseline values according to the following formulas:

The neutrophil-to-lymphocyte ratio (NLR) was calculated as:

NLR = absolute neutrophil count/absolute lymphocyte count
The platelet-to-lymphocyte ratio (PLR) was calculated as:
PLR = platelet count/absolute lymphocyte count
The systemic immune inflammation index (SII) was calculated as:
SII = (platelet count × absolute neutrophil count)/absolute lymphocyte count

All hematological parameters were expressed in ×10^3^/µL and were obtained from the same baseline blood sample.

### 2.4. Outcomes

The primary outcome was the presence of thrombosed hemorrhoids at admission.

Secondary exploratory analyses evaluated:

- the discriminatory performance of NLR, PLR and SII
- trends across NLR tertiles
- associations between inflammatory indices and active hemorrhoidal bleeding

### 2.5. Statistical Analysis

Statistical analysis aimed to compare clinical and laboratory findings between thrombosed and non-thrombosed patients and to identify factors associated with thrombosed hemorrhoids.

Continuous variables were assessed for normality using the Shapiro–Wilk test and are presented as means ± standard deviation (SD) for normally distributed data or medians and interquartile ranges (IQR) for non-normally distributed data. Categorical variables are presented as absolute frequencies and percentages.

Group comparisons were performed using the chi-square test or Fisher’s exact test for categorical variables and the Mann–Whitney U test for continuous variables, as appropriate.

Receiver operating characteristic (ROC) curve analysis was used to evaluate the discriminatory performance of NLR, PLR and SII. The area under the curve (AUC) with 95% confidence intervals (CI) was calculated, and optimal cut-off values were determined using the Youden index.

Univariate logistic regression analysis was initially performed to explore associations between candidate variables and thrombosed hemorrhoids. Variables of clinical relevance were subsequently entered into a multivariable logistic regression model to identify independent predictors. Results are reported as odds ratios (OR) with 95% confidence intervals. A tertile-based trend analysis was additionally performed for NLR. All tests were two-tailed, and a *p*-value < 0.05 was considered statistically significant. Data were initially processed using Microsoft Excel 2016 (Microsoft Corp., Redmond, WA, USA) with XLSTAT 2022 (Addinsoft SARL, Paris, France), and statistical analyses were performed using SPSS version 27.0 (IBM Corp., Armonk, NY, USA).

### 2.6. Ethical Considerations

The study was conducted in accordance with the Declaration of Helsinki and was approved by the Ethics Committee of the County Clinical Emergency Hospital Craiova on 16 January 2026 (approval number: 2136). Given the retrospective design and the use of anonymized data, the requirement for informed consent was waived in accordance with institutional regulations.

## 3. Results

The study cohort included 120 patients, with a predominance of males (67.5%) and a median age of 54 years. The median length of hospital stay was 4 days (Table 1). Regarding hemorrhoid phenotype, internal and mixed forms were most frequently observed. Thrombosed hemorrhoids were identified in 55.8% of patients, while active bleeding was present in 30.8% of cases.

The hematological profile of the cohort indicated median NLR, PLR and SII values consistent with a mild systemic inflammatory status at presentation (Table 2).

Comparative analysis between thrombosed (*n* = 67) and non-thrombosed (*n* = 53) patients showed no statistically significant differences in hematological or inflammatory parameters. Median values of NLR, PLR and SII were comparable between groups (all *p* > 0.05) (Table 3).

Receiver operating characteristic (ROC) analysis demonstrated poor discriminatory performance of inflammatory indices for thrombosed hemorrhoids (Table 4). The AUC values were 0.52 for NLR, 0.55 for PLR, and 0.51 for SII, indicating limited predictive accuracy. None of the evaluated markers reached statistical significance.

In univariate logistic regression analysis, none of the evaluated inflammatory markers were significantly associated with thrombosed hemorrhoids. Age and male sex were also not significant predictors (all *p* > 0.05) (Table 5).

The prevalence of thrombosed hemorrhoids did not show a monotonic pattern across NLR tertiles (52.5%, 65.0% and 50.0% for T1–T3, respectively), and no significant trend was observed (*p* for trend = 0.91) (Table 6).

In multivariable logistic regression analysis adjusted for age, sex and bleeding status, none of the evaluated inflammatory indices (NLR, PLR, or SII) were independently associated with thrombosed hemorrhoids (all *p* > 0.05). Similarly, age, male sex and active bleeding did not emerge as significant predictors in the adjusted model (Table 7).

Inflammatory indices did not differ significantly between patients with and without active bleeding (all *p* > 0.05) (Table 8).

## 4. Discussion

### 4.1. Principal Findings

The present real-world analysis demonstrated that commonly used systemic inflammatory indices—NLR, PLR and SII—have limited utility in predicting thrombosed hemorrhoids. Across multiple complementary statistical approaches, including group comparisons, ROC analysis, tertile trend evaluation and multivariable logistic regression, none of the investigated markers showed significant discriminatory or independent predictive value.

Importantly, ROC analysis revealed AUC values close to 0.5 for all indices, indicating performance near random classification. Specifically, the observed AUC values were 0.52 for NLR, 0.55 for PLR, and 0.51 for SII, all confirming poor discriminatory performance.

Furthermore, the absence of significant associations persisted after adjustment for age, sex and bleeding status, reinforcing the robustness of the negative findings. Notably, the concordant negative results observed across univariate comparisons, ROC analysis, tertile trends and multivariable modeling reduce the likelihood that the findings are attributable to model-specific effects. From a clinical perspective, these results suggest that routine reliance on systemic inflammatory ratios for the identification of thrombosed hemorrhoids is unlikely to provide meaningful incremental value beyond standard clinical assessment.

### 4.2. Comparison with Existing Literature

Systemic inflammatory indices such as NLR, PLR and SII have been widely investigated as inexpensive biomarkers reflecting the balance between pro-inflammatory and immune regulatory pathways. Their prognostic value has been demonstrated in multiple inflammatory and thrombotic conditions, including cardiovascular disease, malignancies and gastrointestinal disorders [8,10,18].

In colorectal and anorectal pathology, prior studies have suggested that elevated inflammatory ratios may correlate with disease severity or acute inflammatory states. For example, research in benign anorectal disease has proposed that NLR may reflect local inflammatory burden and could potentially aid in clinical stratification [1,13,19,20]. Similarly, SII has been increasingly explored as a composite marker integrating neutrophil, platelet and lymphocyte dynamics [21,22].

However, the current findings do not support a meaningful role of these indices in thrombosed hemorrhoids. This discrepancy may reflect important pathophysiological differences. While systemic inflammatory markers perform well in conditions characterized by pronounced systemic inflammation, hemorrhoidal thrombosis is predominantly a localized vascular event with limited systemic inflammatory spillover. Prior mechanistic discussions have emphasized the primarily mechanical and venous stasis–driven nature of hemorrhoidal disease [2,23,24], which may explain the weak systemic signal observed in our cohort.

Thrombosed external hemorrhoids are increasingly described as an acute, predominantly local vascular event characterized by congestion and edema below the dentate line, where management is largely guided by clinical features and timing rather than laboratory markers [25]. In addition, etiological considerations for thrombosed external hemorrhoids include local irritation and inflammatory triggers of the anal skin, further supporting a compartmentalized local mechanism [26].

In contrast, systemic indices such as NLR have shown stronger associations in thrombo-inflammatory conditions with a higher systemic inflammatory burden, including hematologic disorders and broader thrombotic phenotypes [27,28,29]. Taken together, these observations support our results suggesting that routine systemic inflammatory ratios may not capture the biological drivers of hemorrhoidal thrombosis, thereby explaining the near-random ROC performance observed in our cohort.

### 4.3. Interpretation of the Negative Predictive Performance

The near-random AUC values observed in the present study are clinically informative. Rather than indicating methodological failure, they suggest that routine systemic inflammatory indices may not adequately capture the biological processes underlying hemorrhoidal thrombosis. Several pathophysiological and methodological mechanisms may explain this finding.

First, hemorrhoidal thrombosis is primarily an acute localized vascular event with limited systemic inflammatory activation. Experimental and clinical evidence indicates that local venous stasis, vascular congestion and mechanical strain play dominant roles in hemorrhoidal pathology, whereas systemic inflammatory spillover is usually modest [5,30,31,32]. This localized pathophysiology may inherently limit the sensitivity of circulating inflammatory ratios.

Second, the compartmentalized nature of anorectal inflammation may further reduce the diagnostic yield of peripheral biomarkers. In benign anorectal disorders, inflammatory changes are often confined to the local mucosal and vascular microenvironment, with minimal detectable changes in systemic circulation [1,21,33]. Consequently, composite indices derived from peripheral blood counts may underestimate clinically relevant local disease activity.

Third, the magnitude of systemic inflammation in uncomplicated hemorrhoidal disease is typically mild. This is consistent with the relatively low median NLR, PLR and SII values observed in our cohort. Prior investigations have demonstrated that these indices perform optimally in conditions characterized by moderate-to-severe systemic inflammation, such as malignancy, major cardiovascular events, or severe inflammatory disorders [8,10]. In contrast, low-grade inflammatory states may fall below the detection threshold of these composite markers.

Fourth, systemic inflammatory ratios are susceptible to substantial biological variability and multiple confounding influences. Factors such as metabolic status, smoking, occult infections, medication use and age-related immune modulation can significantly affect leukocyte and platelet dynamics independent of anorectal pathology [8,10,34,35]. In heterogeneous real-world populations, this background noise may dilute disease-specific signals and reduce discriminatory performance.

Taken together, these mechanisms provide a biologically plausible explanation for the limited predictive capacity demonstrated in our analysis and suggest that systemic inflammatory indices may have intrinsic limitations in the context of localized hemorrhoidal thrombosis.

From a clinical standpoint, the present findings indicate that routine reliance on NLR, PLR, or SII for risk stratification in thrombosed hemorrhoids is unlikely to provide meaningful added value beyond careful clinical assessment. Local examination and symptom-based evaluation therefore remain the cornerstone of decision-making in this common anorectal condition.

### 4.4. Bleeding Subgroup Findings

Another relevant observation is the lack of significant differences in inflammatory indices between patients with and without active bleeding. Although bleeding may intuitively suggest increased inflammatory activity, hemorrhoidal bleeding is primarily a consequence of mucosal fragility and vascular congestion rather than systemic inflammatory activation.

Prior clinical and pathophysiological descriptions of hemorrhoidal bleeding emphasize its predominantly mechanical and vascular origin, related to dilated hemorrhoidal plexuses and increased venous pressure [1,24]. In this context, bleeding episodes may occur in the absence of a substantial systemic inflammatory response, which is consistent with the comparable NLR, PLR and SII values observed in our cohort.

Furthermore, studies of benign anorectal disease have suggested that local microvascular alterations and connective tissue degeneration play a central role in symptom generation, whereas systemic inflammatory activation remains relatively limited [1,21,36]. This pathophysiological profile may partly explain why composite peripheral inflammatory indices fail to discriminate between bleeding and non-bleeding presentations.

In contrast, in gastrointestinal conditions characterized by overt mucosal inflammation—such as inflammatory bowel disease or advanced colorectal pathology—systemic inflammatory ratios have demonstrated stronger associations with disease activity [14,21,37,38]. The absence of similar findings in hemorrhoidal bleeding further supports the concept that the biological signal in this condition is predominantly local rather than systemic.

Clinically, these findings suggest that the presence of active hemorrhoidal bleeding should not be interpreted as a surrogate marker of systemic inflammatory burden. Instead, clinical evaluation should continue to focus primarily on local examination and symptom severity when guiding management decisions.

### 4.5. Clinical Implications

From a practical standpoint, the present findings have important implications for everyday proctologic practice. First, they suggest that routine calculation of NLR, PLR, or SII for the purpose of predicting thrombosed hemorrhoids is unlikely to provide meaningful clinical benefit. Although these indices have shown prognostic utility in several inflammatory and neoplastic conditions, their performance appears to be context-dependent and closely linked to the magnitude of systemic inflammatory activation [8,10,39,40].

Second, the results caution against overinterpreting mildly elevated inflammatory ratios in patients presenting with hemorrhoidal symptoms. Given the known susceptibility of NLR, PLR and SII to a wide range of physiological and pathological influences—including metabolic factors, subclinical infections and medication effects—isolated elevations may not reflect hemorrhoidal disease activity per se [41,42]. This is particularly relevant in real-world clinical settings, where patient populations are heterogeneous and comorbidity burden is common.

Third, our data support continued reliance on careful clinical examination and symptom assessment as the primary tools for risk stratification in hemorrhoidal disease. Current pathophysiological models emphasize that hemorrhoidal symptoms, including thrombosis and bleeding, are largely driven by local vascular and mechanical mechanisms rather than systemic inflammatory processes [1,43,44]. Accordingly, bedside proctologic evaluation remains the cornerstone of appropriate management. Careful differential diagnosis is essential in patients presenting with acute anorectal pain, as deep anorectal infections may occasionally mimic more benign conditions and require urgent intervention [45].

Importantly, the consistent negative signal observed across multiple analytical approaches—including ROC analysis, subgroup comparisons and multivariable modeling—provides useful evidence helping to refine the role of systemic biomarkers in benign anorectal pathology. Rather than supporting routine use, the present findings suggest that inflammatory ratios should be interpreted cautiously and within the broader clinical context.

From a health systems perspective, avoiding unnecessary reliance on low-yield biomarkers may also contribute to more cost-effective and streamlined evaluation pathways in patients with suspected hemorrhoidal disease.

### 4.6. Strengths and Limitations

The present study has several important strengths. First, it reflects a real-world clinical cohort with a balanced distribution of thrombosed and non-thrombosed cases, enhancing the clinical relevance of the findings. Second, multiple complementary statistical approaches were applied—including ROC analysis, tertile trend evaluation and multivariable logistic regression—which strengthens the internal consistency and robustness of the results. Third, the simultaneous evaluation of NLR, PLR and SII provides a comprehensive assessment of widely used systemic inflammatory indices within the same clinical framework, addressing a gap in the current literature.

Importantly, the study contributes clinically meaningful negative evidence, demonstrating that these readily available inflammatory ratios show near-random discriminatory performance in thrombosed hemorrhoids. In a research field often affected by publication bias toward positive biomarkers, such findings help refine the appropriate clinical role of systemic inflammatory indices and may prevent their overinterpretation in benign anorectal disease.

Several limitations should nevertheless be acknowledged. First, the retrospective single-center design introduces the possibility of selection bias and may limit external generalizability. Second, although the cohort included 120 patients, the sample size may still be insufficient to detect small effect sizes, as suggested by the relatively wide confidence intervals observed in the adjusted models. Therefore, a modest association cannot be completely excluded.

Third, only baseline inflammatory markers were analyzed. Because systemic inflammatory indices may exhibit temporal variability, the lack of serial measurements may have limited the ability to capture dynamic inflammatory changes surrounding the thrombotic event. Fourth, despite multivariable adjustment, residual confounding from unmeasured factors—such as subclinical infections, metabolic status, medication use, or smoking—cannot be fully ruled out in this real-world population. Although major confounding conditions such as active infection, inflammatory diseases, hematologic malignancies and immunosuppressive therapy were excluded, detailed information on other comorbidities (e.g., metabolic conditions, smoking status, or subclinical inflammatory states) was not consistently available and may have influenced inflammatory indices.

In addition, systemic inflammatory indices such as NLR, PLR and SII may be influenced by factors unrelated to hemorrhoidal pathology, including the use of anticoagulant or antiplatelet therapy and variations in bleeding severity. These factors may affect hematological parameters and may represent additional sources of residual confounding. Unfortunately, detailed information regarding anticoagulant use was not consistently available in this retrospective dataset [46,47].

Another limitation is the lack of standardized grading of hemorrhoidal disease severity (e.g., Goligher classification). Therefore, the potential impact of disease stage on thrombosis and inflammatory indices could not be evaluated.

Finally, external validation in independent and geographically diverse cohorts was not performed. Prospective multicenter studies will be necessary to confirm the generalizability of the present findings and to determine whether specific patient subgroups might still derive incremental value from systemic inflammatory markers.

### 4.7. Future Directions

Future research should focus on prospective multicenter cohorts with standardized timing of laboratory measurements and larger sample sizes to improve statistical power and external validity. Given the relatively modest systemic inflammatory signal observed in hemorrhoidal disease, studies incorporating longitudinal biomarker dynamics rather than single baseline measurements may provide additional insight into temporal inflammatory patterns.

Importantly, future investigations should also explore local inflammatory markers and tissue-level biomarkers, which may better reflect the compartmentalized pathophysiology of hemorrhoidal disease. Experimental and clinical data increasingly suggest that benign anorectal disorders are driven predominantly by local vascular, connective tissue and microinflammatory changes rather than systemic immune activation [1,8]. Accordingly, analyses focusing on mucosal cytokine profiles, local endothelial dysfunction, or microvascular alterations may yield more sensitive disease-specific indicators.

In addition, integration of systemic indices with clinical scoring systems or imaging-based parameters may improve risk stratification compared with isolated hematological ratios alone. Prior biomarker research in gastrointestinal and inflammatory conditions has demonstrated that multimodal approaches often outperform single laboratory parameters [10,21,48].

Finally, external validation in independent and geographically diverse cohorts will be essential to determine the generalizability of the present findings and to clarify whether specific patient subgroups might still derive limited benefit from inflammatory ratio assessment.

In addition, integrating clinical severity scores with laboratory parameters and imaging findings may help develop more accurate risk stratification tools for hemorrhoidal disease.

## 5. Conclusions

In this real-world cohort of patients with hemorrhoidal disease, systemic inflammatory indices—including NLR, PLR and SII—showed consistently poor diagnostic and predictive performance for thrombosed hemorrhoids. These findings suggest that routinely used inflammatory ratios provide limited incremental value beyond standard clinical assessment in this setting. Importantly, the results highlight potential intrinsic limitations of systemic biomarkers in predominantly localized benign anorectal conditions. Prospective multicenter studies exploring longitudinal and tissue-level markers are warranted to identify more sensitive indicators of hemorrhoidal thrombosis.

## Figures and Tables

**Table 1:** Baseline and clinical characteristics of the study cohort (*N* = 120).

Characteristic	Value
***N* (patients)**	120
**Sex, *n* (%)**	
**Male**	81 (67.5%)
**Female**	39 (32.5%)
**Age, years, median (IQR)**	54.00 (43.00–61.50)
**Length of hospital stay, days, median (IQR)**	4.00 (2.75–5.00)
**Hemorrhoid phenotype, *n* (%)**	
**Internal**	49 (40.8%)
**External**	25 (20.8%)
**Mixed**	46 (38.3%)
**Thrombosed hemorrhoids, *n* (%)**	
**Yes**	67 (55.8%)
**No**	53 (44.2%)
**Bleeding hemorrhoids, *n* (%)**	
**Yes**	37 (30.8%)
**No**	83 (69.2%)

*Note*: IQR—interquartile range.

**Table 2:** Hematological parameters and inflammatory indices at presentation.

Parameter	Median (IQR)
**Hemoglobin (g/dL)**	13.40 (11.54–14.55)
**Hematocrit (%)**	41.55 (33.74–44.25)
**White blood cells (×10^3^/µL)**	7.28 (5.90–9.55)
**Neutrophils (×10^3^/µL)**	4.44 (3.20–6.31)
**Lymphocytes (×10^3^/µL)**	1.90 (1.60–2.32)
**Monocytes (×10^3^/µL)**	0.52 (0.40–0.68)
**Platelets (×10^3^/µL)**	231.00 (201.43–282.75)
**NLR**	2.35 (1.77–3.08)
**PLR**	139.06 (100.24–163.09)
**MLR**	0.26 (0.21–0.38)
**SII**	537.66 (381.66–755.36)

*Note*: NLR—neutrophil-to-lymphocyte ratio; PLR—platelet-to-lymphocyte ratio; MLR—monocyte-to-lymphocyte ratio; SII—systemic immune inflammation index. Values are presented as median and interquartile range (IQR).

**Table 3:** Exploratory comparison: thrombosed vs. non-thrombosed hemorrhoids.

Parameter	Non-Thrombosed (*n* = 53) Median (IQR)	Thrombosed (*n* = 67) Median (IQR)	*p*-Value ^†^
**Hemoglobin (g/dL)**	13.40 (11.68–14.50)	13.70 (10.80–14.95)	0.715
**Hematocrit (%)**	41.30 (33.77–44.60)	42.53 (33.49–43.80)	0.946
**White blood cells (×10^3^/µL)**	6.87 (5.47–9.47)	7.71 (6.49–10.34)	0.354
**Neutrophils (×10^3^/µL)**	4.23 (3.20–6.43)	4.87 (3.46–5.05)	0.926
**Lymphocytes (×10^3^/µL)**	1.90 (1.60–2.34)	1.96 (1.61–2.08)	0.926
**Platelets (×10^3^/µL)**	231.00 (202.40–285.00)	212.00 (167.10–270.00)	0.666
**NLR**	2.37 (1.77–3.06)	2.34 (1.77–3.13)	0.950
**PLR**	139.95 (104.28–161.81)	101.92 (85.30–180.00)	0.730
**SII**	543.28 (388.14–707.44)	496.37 (295.47–1168.70)	1.000

*Note*: IQR—interquartile range; NLR—neutrophil-to-lymphocyte ratio; PLR—platelet-to-lymphocyte ratio; SII—systemic immune inflammation index. ^†^ Mann–Whitney U test (two-sided). Effect size interpreted as: small (0.1), moderate (0.3), large (0.5).

**Table 4:** Diagnostic performance of inflammatory indices for thrombosed hemorrhoids (*N* = 120).

Marker	AUC (95% CI)	Optimal Cut-Off	Sensitivity (%)	Specificity (%)	*p*-Value
**NLR**	0.52 (0.36–0.68)	2.45	46.2	57.0	0.78
**PLR**	0.55 (0.39–0.70)	135.0	53.8	55.1	0.61
**SII**	0.51 (0.35–0.67)	540	46.2	56.1	0.92

*Note*: AUC—area under the curve; CI—confidence interval; NLR—neutrophil-to-lymphocyte ratio; PLR—platelet-to-lymphocyte ratio; SII—systemic immune inflammation index. AUC values were derived from ROC curve analysis; optimal cut-off values were determined using the Youden index.

**Table 5:** Logistic regression analysis for predictors of thrombosed hemorrhoids (*N* = 120).

Variable	OR	95% CI	*p*-Value
**Age (per year)**	1.01	0.98–1.04	0.48
**Male sex**	1.12	0.39–3.21	0.83
**NLR**	1.05	0.78–1.41	0.74
**PLR**	1.00	0.99–1.01	0.69
**SII**	1.00	0.99–1.00	0.88

*Note*: OR—odds ratio; CI—confidence interval; NLR—neutrophil-to-lymphocyte ratio; PLR—platelet-to-lymphocyte ratio; SII—systemic immune inflammation index. Logistic regression was performed using univariate models.

**Table 6:** Trend analysis across NLR tertiles (*N* = 120).

NLR Tertile	Patients (*n*)	Thrombosis *n* (%)
**T1 (low)**	40	21 (52.5%)
**T2 (middle)**	40	26 (65.5%)
**T3 (high)**	40	20 (50.0%)

*Note*: Trend test *p* = 0.91.

**Table 7:** Multivariable logistic regression for predictors of thrombosed hemorrhoids (*N* = 120).

Variable	aOR	95% CI	*p*-Value
**Age (per year)**	0.99	0.95–1.03	0.692
**Male sex**	0.61	0.19–1.89	0.390
**NLR (per 1 unit)**	1.27	0.70–2.29	0.435
**PLR (per 10 units)**	1.04	0.89–1.20	0.651
**SII (per 100 units)**	0.92	0.69–1.23	0.594
**Bleeding (yes vs. no)**	1.51	0.67–3.40	0.324

*Note*: aOR—adjusted odds ratio; CI—confidence interval; NLR—neutrophil-to-lymphocyte ratio; PLR—platelet-to-lymphocyte ratio; SII—systemic immune inflammation index. PLR was modeled per 10-unit increase and SII per 100-unit increase to improve interpretability. The multivariable model included age, sex, NLR, PLR, SII and bleeding status.

**Table 8:** Comparison of inflammatory indices according to bleeding status.

Parameter	No Bleeding (*n* = 83) Median (IQR)	Bleeding (*n* = 37) Median (IQR)	*p*-Value ^†^
**NLR**	2.33 (1.75–3.05)	2.41 (1.82–3.12)	0.680
**PLR**	137.8 (99.5–161.2)	141.6 (102.3–166.4)	0.740
**SII**	528.4 (375.2–742.1)	556.3 (392.8–781.5)	0.620

*Note*: Values are presented as median and interquartile range (IQR). NLR—neutrophil-to-lymphocyte ratio; PLR—platelet-to-lymphocyte ratio; SII—systemic immune inflammation index. ^†^ Mann–Whitney U test (two-sided).

## Data Availability

The data presented in this study are available on request from the corresponding author. The data are not publicly available due to patient confidentiality.
